# Proliferating Cell Nuclear Antigen in the Era of Oncolytic Virotherapy

**DOI:** 10.3390/v16081264

**Published:** 2024-08-07

**Authors:** Amy Kwan, India Mcdermott-Brown, Munitta Muthana

**Affiliations:** Medical School, University of Sheffield, Beech Hill Road, Sheffield S10 2RX, UK; amy.kwan@nhs.net (A.K.);

**Keywords:** PCNA, oncolytic virotherapy, cancer, immune cells

## Abstract

Proliferating cell nuclear antigen (PCNA) is a well-documented accessory protein of DNA repair and replication. It belongs to the sliding clamp family of proteins that encircle DNA and acts as a mobile docking platform for interacting proteins to mount and perform their metabolic tasks. PCNA presence is ubiquitous to all cells, and when located in the nucleus it plays a role in DNA replication and repair, cell cycle control and apoptosis in proliferating cells. It also plays a crucial role in the infectivity of some viruses, such as herpes simplex viruses (HSVs). However, more recently it has been found in the cytoplasm of immune cells such as neutrophils and macrophages where it has been shown to be involved in the development of a pro-inflammatory state. PCNA is also expressed on the surface of certain cancer cells and can play a role in preventing immune cells from killing tumours, as well as being associated with cancer virulence. Given the growing interest in oncolytic viruses (OVs) as a novel cancer therapeutic, this review considers the role of PCNA in healthy, cancerous, and immune cells to gain an understanding of how PCNA targeted therapy and oncolytic virotherapy may interact in the future.

## 1. Introduction

**Figure 1:**
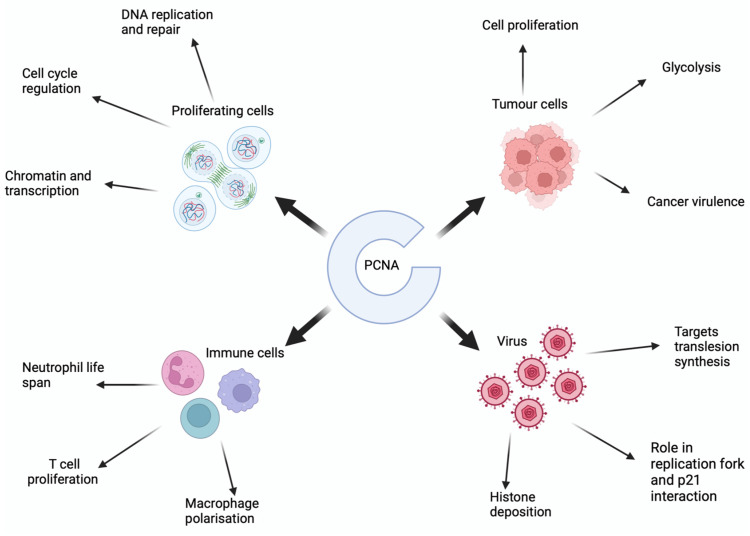
The many roles of PCNA within healthy and diseased cells.

PCNA is a well-documented accessory protein of DNA repair, replication, and recombination [1]. It belongs to the sliding clamp family of proteins, which are toroidal shaped, six-domain processivity factors that encircle DNA and act as a mobile docking platform for interacting proteins to mount and perform metabolic tasks [2,3]. This function, within healthy proliferating cells, is highly documented and understood. However, its function within diseased cells, whether cancerous or virally infected, has led to an increased interest in PCNA over the recent years. Given its role in DNA repair, the relationship with PCNA and cancer cells is thought to play a key role in both carcinogenesis and subsequent cancer growth and metastases. Although PCNA is present in all cells, it exists as three main subtypes, with differences in the quantity of expression in cancer cells [4]. Indeed, PCNA demonstrates similar post-translational modifications between normal and cancer cells, with the only significant difference being that the protein level of PCNA is several-fold higher in cancer cells [4]. Malkas and colleagues have documented a cancer-specific isoform of PCNA in breast cancer cells that interacts with DNA polymerase δ [5] They have developed a polyclonal antibody specific to this isoform, and they suggest this antibody may serve as a biomarker of breast cancer through histological staining. However, further investigations are required to fully test the sensitivity of this antibody with commercially available PCNA antibodies [4] and determine which cell types within the tissue express PCNA, as well as ascertain expression of this isoform across a larger patient cohort and in different breast cancer subtypes. Nevertheless, there have been recent phase 1 clinical trials of a targeted therapy to one of the PCNA isoforms [5,6,7] and other PCNA targeted therapies are in development. Given PCNA can also be hijacked by viruses to aid in viral replication and virotherapy, and correspondingly is a growing area of oncological treatments, it was timely that our group have shown that PCNA within macrophages is needed to illicit the immune-mediated response to oncolytic virotherapy [8]. Considering these developments, this review explores the role of PCNA in healthy cells, virally infected cells, immune cells, and cancerous cells to gain an understanding of how PCNA targeted therapy and oncolytic virotherapy may interact in the future. These multifaceted roles are summarized in Figure 1.

## 2. The Structure of PCNA

PCNA forms a unique structure called a sliding clamp which allows it to embrace DNA and slide up and down strands. It comprises three identical monomeric units, all of which join in a head-to-tail configuration to form a homotrimeric ring. The inner surface consists of positively charged alpha-helices and the outer surface consists of beta-sheets and inter-domain connecting loops (IDCL) which serve as a docking site for proteins, including p21 and polymerase (Pol) δ [1]. PCNA acts as a mediator between the interacting protein and DNA, performing a tethering action which stabilizes interactions and prevents the dissociation of interacting proteins [9].

The major interaction sites on PCNA are IDCL, N-terminal, and C-terminal. Proteins paramount to cellular replication and function adhere to these sites and subsequently perform their metabolic function [1]. Most bindings contain either a KA-box or PCNA-interacting peptide (PIP)-box motif, the latter of which has the consensus sequence Q-x-x-(h)-x-x-(a)-(a), where x represents any residue, h represents residues with hydrophobic, aromatic side chains, and a represents hydrophobic residues [1,3]. These are common molecular patterns detectable by PCNA. Some PCNA binding proteins are capable of binding without these motifs. Likewise, proteins with a low affinity to PCNA become potential binding partners when levels of both are high [10].

**Figure 2:**
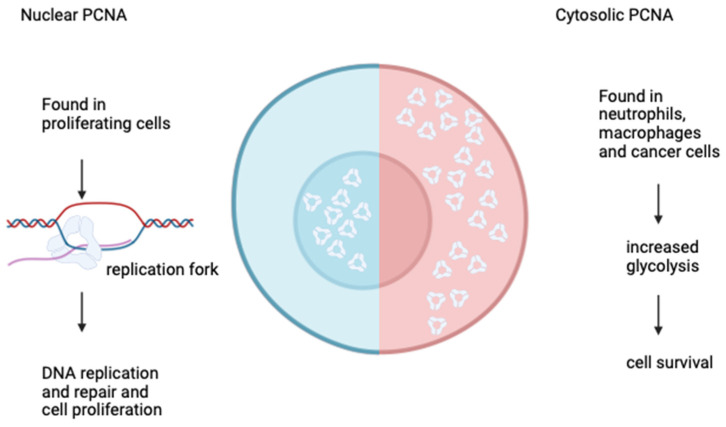
Location of PCNA within cells. The role of PCNA is dependent on location and cell type. Proliferating cells demonstrate higher amounts of PCNA within the nucleus leading to cell division. However, more mature cells/cancer cells show an increase in the cytoplasm, which leads to prolongation of cell survival.

PCNA exists in at least three main isoforms (main, acidic form, and basic form) which increase through the cell cycle, coinciding with DNA replication activity [11]. PCNA can exist within the nucleus or the cytoplasm of cells and the function of PCNA is dependent on its home environment (see Figure 2). The extensive work by Naryzhny and Lee has contributed significantly to the understanding of the various isoforms of PCNA. Of note are the characteristics of the three main isoforms and their presence and role and acetylation status with normal cells [4]. Here, a moderately acetylated main (M) form was found in all the subcellular compartments of cycling cells, whereas a highly acetylated acidic form was primarily found in the nucleoplasm, nuclear matrix, and chromatin with a deacetylated basic form was most pronounced in the cytoplasm of cycling cells. In proliferating cells, PCNA’s role within the replication fork can be highjacked by viral pathways and used to enable viral replication; however, the presence of PCNA within neutrophils may allow for an enhanced immune system response to viral infection. These complex, and sometimes contradictory, roles are discussed further below.

## 3. What Is the Role of PCNA in Eukaryotic Cells?

The role of PCNA within eukaryotic cells has been discussed in other reviews where its function has been categorised as “maestro of the replication fork”. Here, it performs roles within DNA replication and repair, cell cycle regulation [1], sister chromatid cohesion [12], and chromatin assembly [13]. The most well-documented function of PCNA is as a processivity factor during the replication of DNA. This process starts when PCNA is loaded onto the genomic template by replication factor C and reforms a homotrimer around DNA [14]. The basic residues lining the inside of PCNA form polar bonds with the phosphate backbone of DNA. These bonds are then sequentially broken and reformed propelling the PCNA along the helix [15]. As PCNA shafts along the DNA, leading strand synthesis occurs in a continuous manner catalysed by Polε. On the lagging strand, the discontinuous synthesis (due to the limitation of printing with 5′-3′ directionality) is performed by Polδ [16]. The Okazaki fragments are then processed by FEN1 and LIG1 which likewise rely on PCNA mediation [1,14,17].

For DNA replication to be executed to a high fidelity, DNA damage is continually repaired to prevent delays in replication fork progression and the induction of double strand breaks and fork collapse [18], and this is aided by post-translational mono-ubiquitination, polyubiquitination, and sumolyation of PCNA. Mono-ubiquitination of PCNA at Lysine 164 occurs in response to halting of the replication fork and recruits DNA polymerase to commence the translesion synthesis (TLS) process [19]. This enables a replicative polymerase to resume replication from the newly incorporated nucleotide [19]. Polyubiquitination of PCNA facilitates error-free lesion bypass and restarts the replication fork after halting and sumolyation of PCNA prevents the cohesion and recombination of sister chromatids during the S phase of the cell cycle [18,20].

It is evident that PCNA participates in numerous fundamental processes in the healthy cell; however, there still stands the unresolved issue of how the interactions are coordinated. Some models suggest they are regulated through differential bonding affinities [3,21]. However, this model does not account for the complexity of the molecule; PCNA consists of three identical monomeric units and can therefore bind numerous molecules at once, complicating the process [21]. A number of alternative models have been suggested. Prosperi proposed an alternative “dynamic hand-off” or “passing the baton” model where the substrate of every metabolic step facilitates the binding of the next molecule [22]. Naryzhny has suggested that PCNA may function as a double homotrimer complex as this allows simultaneous accommodation of large proteins such as chromatin assembly factor-1 and pol δ that are pertinent to DNA replication and repair pathways [23]. Additionally, recent Cryo-EM snapshots have shown a structure which suggests a continuous DNA-binding surface is formed between DNA ligase and PCNA that supports the distorted conformation of a DNA break and contributes to PCNA stimulation of DNA ligation [24]. This may provide the framework to future work facilitating the coordination of multiple enzymes in DNA replication or repair [24,25]. However, there remains room for either confirmation or disregard of these models through further research.

## 4. What Is the Role of PCNA in Cancer?

Within cancer cells, PCNA can be found on the cell surface, in the cytoplasm, and the perinuclear area. To gain insight into cytoplasmic PCNA–protein interactions, Western blotting and mass spectrometry have been used to identify PCNA partners [10]. Here, Naryzhny suggested that PCNA–protein interactions may differ between cancerous cells (MDA-MB468) and non-cancerous cells (184B5). PCNA was found to interact with six glycolytic enzymes involved in the sequential steps 4–9 of glycolysis in cancerous cells through their KA-box motif. While the levels of these proteins did not differ between the cancerous and non-cancerous cells, there was evidence of discrepancies between the post-translational modifications of glyceraldehyde-3-posphate and phosphoglycerate kinase 1 between the cancerous and non-cancerous cells. Naryzhny also proposed these discrepancies may contribute to regulation of PCNA–protein interactions through altering protein affinity for PCNA. These findings support the theory that PCNA participates in glycolysis in cancerous cells through not only interacting with glycolytic enzymes in stages 4–9 of glycolysis, but also via upregulating glycolysis though an interaction with NAMPT within chemotherapy resistant cells. Glycolysis is one of the main energy generating pathways in cells, thus increased gliosis is likely to cause increase in cancer growth. Along with evidencing PCNA’s involvement in glycolysis, the authors found PCNA interacted with several cytoplasmic oncoproteins which were upregulated in the cancerous cells including malate dehydrogenase (which helps generate cytosolic NAD in cancer cells and activated primary T cells), eEF2K (which helps prime the TME and recruits TAMs), and peptidyl-prolyl isomerase (PIN1 which is involved in the expansion of cancer stem cells). This suggests that although PCNA is present in all cells, PCNA has an important role in oncogenic regulation and development within cancer cells.

In addition, another study [26] found an association between chemotherapy resistance, cancer cell survival, and PCNA. In Daunorubicin chemotherapy resistant HL-60 cells (HL-60R), there was significant pooling of PCNA within the cytoplasm of the cancerous cells compared the Daunorubicin sensitive counterpart cells (HL-60S). The PCNA pooling occurred because of increased nuclear export from overexpression of CRM-1. It was found that an interaction between PCNA and nicotinamide phosphoribosyltransferase (NAMPT)—a mediating protein in the biosynthesis of nicotinamide adenine dinucleotide (NAD) [27]–coordinated cell survival and glycolysis within the cancerous cells. The reaction between NAMPT and PCNA prompted an increase in the concentration of NAD+, expression of hexokinase 1, and lactate production, which potentiated the glycolytic pathway and facilitated cell survival associated with chemotherapy resistance [28]. Ohayon and co-workers proposed that cytosolic PCNA in HL-60R cells builds a protein scaffold which possesses the ability to mediate cell survival through increasing glycolytic metabolism [26].

## 5. What Is Oncolytic Virotherapy?

An oncolytic virus is one that has the ability, either intrinsically, but more commonly genetically altered, to preferentially divide and replicate in cancer cells rather than non-cancer cells. Although the mechanism of how individual viruses deliver their cytotoxic effect differs, the broad effect of the virus is to directly lyse tumour cells and generate immunogenic cell death [29,30,31], thus releasing tumour antigens, which will be recognized and targetable by the hosts own immune system. Immunogenic cell death is the term given to the release of a host of pro-inflammatory markers including calreticulin, heat shock proteins, ATP, and HMGB1. This environment is particularly appealing to dendritic cells which then phagocytose tumour-associated antigens and present these to T cells.

The most studied of these are adenoviruses, reoviruses, and herpes simplex viruses (HSVs). These have been trialled in several tumour types with preclinical and clinical efficacy, particularly in advanced melanoma with current FDA/EMA approval of the first FDA approved oncolytic virus Talimogene laherparepvec (T-VEC). T-VEC is a modified HSV-1 that carries the transgene for GM-CSF. The landmark phase 3 virotherapy trial (OPTIM) described an increased response and overall survival in patients treated with this in comparison with GM-CSF alone [32]. This virus, and many others, are given intratumourally due to the challenges of systemic therapy. There is ongoing study into oncolytic virotherapy, which include the identification of a virus which can be delivered systemically without a vector (stability in blood), genetically changing a virus so it targets cancer cells more specifically to generate an appropriate downstream immune response and trying to enhance response of virotherapy with combination of chemotherapy and other anticancer treatments [33,34].

PCNA is often needed for oncolytic viral replication. Although present in all cell types, it has been found to be overexpressed in most cancers and has been speculated to aid the efficacy of oncolytic virotherapy. Adeno-associated virus 2 requires replication protein A (RPA), replication factor C (RFC), proliferating cell nuclear antigen (PCNA), and DNA polymerase delta (POLD1) to be present for replication, and this specific combination of factors has been found to be present in cervical cancer cells [35]. Interestingly PCNA may also play a role in cells of the tumour microenvironment. When PCNA was knocked down within monocyte derived macrophages and then infected with HSV1716 in the presence of triple negative breast cancer (TNBC) cells, it was found that the presence of PCNA is required for HSV replication [8]. Given the growing interest in oncolytic virotherapy, the role of PCNA in viral replication and immune cell function, particularly with reference to HSV, is discussed below.

## 6. What Is Role of PCNA in Viral Replication?

Given PCNA’s role in DNA replication and the cell cycle within normal eukaryotic cells, it is unsurprising that PCNA has been shown to be involved in the replication of several DNA viruses. For example, in the vaccinia virus, from the poxvirus family, PCNA has been found to be required for the viral replication and inhibition of PCNA reduced genome replication and late viral protein expression [36]. Furthermore, in a non-cancer setting, a study found that HSV targets the translesion synthesis (TLS) pathway of DNA damage-tolerance within cells to facilitate its own proliferation [37]. In this pathway, as previously explained, PCNA is ubiquitinated following stalling of the replication fork. Dong et al. found that HSV-1 deubiquitinase UL36USP deubiquitinated PCNA, inhibiting the formation of pol δ foci following DNA-damage, thereby suppressing the TLS pathway. In order to facilitate its infectivity and overcome cellular defence mechanisms, it was suggested that HSV inactivates the TLS process and recruits PCNA to facilitate its own replication.

Another study documented PCNA involvement in histone deposition during HSV-1 viral proliferation [38]. The HSV-1 virus replicates using its own processivity factors (UL30 and UL42), and work by Sanders et al. suggested PCNA was not involved in viral DNA-replicating, but rather in another stage of the viral growth cycle. Following PCNA siRNA knockdown, the histone deposition on the HSV-1 genome process did not occur; in this experiment, PCNA’s involvement in histone deposition was dependent on an interaction with chromatin assembly factors Asf1 and CAF-1. This corresponds with similar findings that HSV growth and replication requires another factor, Asf1 [39].

PCNA is one of the most enriched proteins on the HSV-1 replication fork [40] and studies have shown that PCNA inhibition using the small molecular inhibitor PCNA-I1 reversibly inhibits HSV-1 DNA replication [41]. To illustrate this, Packard et al. inhibited PCNA function with two different PCNA inhibitors; PCNA-I1 and T2AA. PCNA-I1 stabilizes the PCNA homotrimer and reduces the repair of double strand breaks by homologous recombination and suppresses nucleotide excision repair [42]. T2AA inhibits interactions between proteins that contain a PCNA interacting protein (PIP)-box motif and the PCNA IDCL or mono-ubiquitinated K164 [43]. Here, they show that PCNA must function at a few levels, as despite PCNA inhibitors causing decrease viral replication, they also result in decrease late gene expression and abnormal virus production. Authors concluded that in addition to anti-cancer treatments, perhaps targeting PCNA may provide a novel was of treating HSV-1 infection [41].

However, this may not be the whole picture. Indeed, PCNA’s role in HSV pathogenesis and replication is of interest to us as we found that knockdown of PCNA within TAMs decreased the effectiveness of the oncolytic virus HSV1716, a strain derived from HSV-1. HSV1716 has a deletion of the viral neurovirulence factor ICP34.5. ICP34.5 has been shown to interact with the viral replication proteins UL30, UL42, and PCNA within the replication fork, and given we have shown HSV1716 viral replication can only happen when PCNA is present (but not absent) despite the deletion ICP34.5, this interaction does not provide the complete mechanism [8]. PCNA presence in clinical patients receiving HSV1716 further supports a relationship between HSV oncolytic virotherapy and PCNA. Histological staining of tumour sections from an early phase clinical trial, after treatment with HSV1716, revealed expression of PCNA positive cells. This included ten metastatic tumours (three melanoma, four carcinoma, and three adenocarcinoma) as well as four glioblastoma multiforme and the commercially available antibody, Clone PC10 [44]. This provides an interesting documentary of PCNA presence; however, the effect of PCNA inhibition on oncolytic virotherapy is uncertain.

Within a cancer setting, some oncolytic viruses indirectly inhibit p21 by targeting p53 [45], thus the role of p21 and PCNA has been questioned. p21 has been shown to inhibit parvovirus replication in vitro, and this effect was shown to be reversed by the addition of PCNA [46]. Furthermore, when investigating the role of p21 in parvoviruses, PCNA was found to provide a molecular platform for p21 to be recognized by E3-ubiquitin ligase, CRL4Cdt2, and subsequent p21 depletion prevented the interaction with PCNA which led to the inhibition of efficient viral replication [47]. As PCNA plays a vital role in viral replication, the interactions with p21 in the context of viral infection may emerge as an important tool for allowing sustained viral replication.

## 7. How Does PCNA Regulate Immune Cell Functioning?

**Figure 3:**
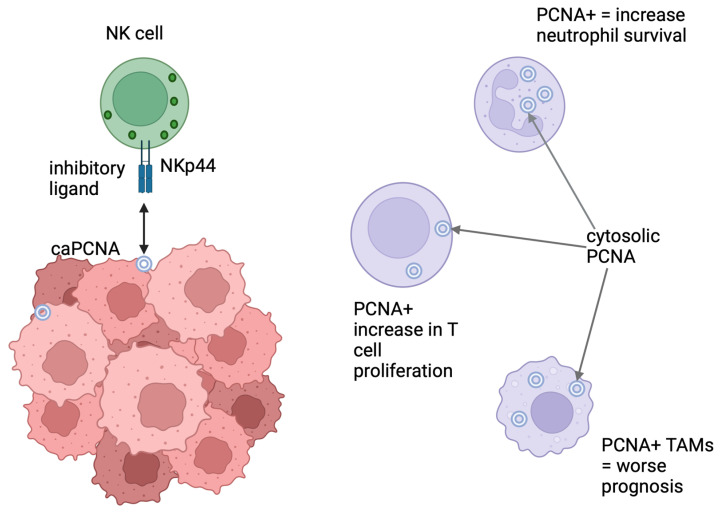
PCNA and its role in the tumour microenvironment. PCNA is present on the cell surface of cancer cells and its role is to enhance immune evasion by prevention of NK cell activation and degranulation through the inhibitory receptor NKP44. PCNA is found in the cytosol of neutrophils, T cells, and tumour-associated macrophages. In neutrophils, the function of PCNA is to prolong survival through modulation of glycolysis. In TAMs, the function of PCNA is unknown; however, these TAMs express more M1-like markers which are normally associated with cancer cytotoxicity. Interestingly a higher prevalence of PCNA+ TAMs suggests poorer prognosis, and further studies are needed to understand these findings. Little information is available about lymphocytes, although the role of PCNA in the proliferation of T cells is documented.

PCNA is a primary nuclear protein, but some studies indicate its presence in the cytoplasm, where it has been proposed to regulate neutrophil lifespan [48], or on the cell surface, where it acts as a ligand for the natural cytotoxicity receptor NKp44 to regulate the activation of natural killer cells [49]. In T-cell lymphomas (Sezary syndrome), PCNA was found to be expressed on cancerous T cells and blockade of PCNA using the antibody-mAb14-enhanced NK-mediated lysis of these cells [50,51]. The mechanism behind the presentation of PCNA on the cell surface is not fully clear; there is a suggestion that extracellular vesicles can transfer PCNA between cells [52]. Here, Lei et al. showed that neonatal umbilical cord bone marrow derived stem cells receive PCNA from extracellular vesicles and this inhibits bone and kidney degeneration associated with ageing. Understanding the difference in isoforms and location of PCNA in different cell types may explain the role of PCNA within the tumour microenvironment (Figure 3).

### 7.1. Neutrophils

PCNA’s functions in neutrophils are well documented, with the literature evidencing its involvement in neutrophil survival mechanisms. Following engulfment and degradation of pathogens, neutrophils undergo apoptosis and phagocytosis from macrophages [53]. Failure to undergo apoptosis causes neutrophilia, a disease associated with hypoxia, neurological deficits, and dyspnea; accelerated apoptosis/deficit causes neutropenia, increasing infection susceptibility to a site of injury [54], therefore neutrophils require a tightly controlled lifespan.

Despite neutrophils being terminally differentiated and unable to proliferate, they express large amounts of exclusively cytosolic PCNA. The relocation of PCNA from nucleus to cytoplasm occurs during granulocytic differentiation [55]. Here, the cytosolic PCNA is evidenced to play an anti-apoptotic role in neutrophils [48]. Through sequestering intracellular procaspase 3, procaspase 8, procaspase 9, and procaspase 10, PCNA prevents their activation. Activation of these molecules occurs via cleavage into caspases; the caspases then proceed to execute cell apoptosis [56]. Sequestration of procaspases by PCNA therefore prevents apoptosis of the cell [48,57] and demonstrated that p21 triggers the degradation of PCNA during neutrophil apoptosis, thereby removing PCNA’s anti-apoptotic role enabling the cell apoptotic process [53].

Neutrophil release from the bone marrow can be stimulated by G-CSF. On administration of G-CSF, the PCNA scaffold is altered, which allows interaction with glycolytic enzymes. This allows PCNA to govern neutrophil functionality as well as prolonged survival which may be instrumental in an inflammatory setting in response to a pathogen or malignancy [58]. Furthermore, in an activated state, for example infection with COVID-19, PCNA was found to be elevated in the cytosol of patients with active infection [59]. Authors also found an associative increase in the protein S100A8, which is known to induce neutrophil chemotaxis and migration. Authors suggest that a PCNA-S100A8 complex acts as potential driver for neutrophil dysregulation (as observed in COVID-19) and a decisive component of both neutrophil activation and heterogeneity. Given the role of PCNA to prolong neutrophil survival and contribute to the neutrophil dominant states (infection or acute inflammation), it has been of increasing interest to explore the targeting of PCNA to reduce long term damage and the development of chronic inflammation [60]. At the present, the clinical application of this is limited; however, given the availability PCNA inhibitors that have been described in recent years, further studies in this area are expected.

### 7.2. NK Cells

NK cells are the effector lymphocytes of the innate immune system and are responsible for limiting the initial phase of inflammation and infection [61]. They can be activated through the stimulation of the NK receptor. PCNA is expressed on the surface of cancer cells and acts as an inhibitory ligand for the NK-cell receptor, NKp44-isoform1 [62]. Garzetti et al. have shown an inverse relationship between the ability of a natural killer cell to target cancer cells and the PCNA quantity within cancer cells; however, only association, not causation, had been determined [63]. NKp44 receptor is an activating receptor on natural killer cells which, when activated, stimulates the natural killer cell and its lysing properties. Rosental et al. found PCNA became cell membrane localized following engagement with natural killer cells [62]. Here, it functions as an inhibitory ligand for the NKp44 receptor, reducing the receptor’s ability to activate. PCNA did not act by preventing interaction between NKp44 and activating ligands, rather in a means which produced an inhibitory signal mediated by NKp44-immunoreceptor tyrosine-based inhibitory motif. By inhibiting the NKp44 receptor, PCNA facilitates tumour immune evasion promoting tumour survival. This study is effective in highlighting PCNA’s function as a ligand, intervening in immunological processes alongside its well evidenced role as a processivity factor/coenzyme. With this knowledge, Kundu et al. have subsequently reported the formation of a mAb against human PCNA, 14-25-9, that recognizes cell surface PCNA on solid and leukemic cancer cell lines, as well as tumour cells from PDXs and blocks the NKp44-PCNA IC [51]. mAb 14-25-9 was shown to increase functional activity of both human primary NK cells and the NK-92 cell line overexpressing NKp44-Iso1, and can suppress tumour growth in PDX-bearing NSG mice. Within breast cancer, similar interactions between PCNA and NKp44 have been observed. Furthermore, PCNA has also been detected on the cell surface on a number of a number of TNBC cell lines, and Marrufo et al. found that inhibition of PCNA with antibodies disrupted interaction with NKp44 and enhanced lysis by primary NK cells [64]. With these results they concluded that the presence of cell surface PCNA enabled immune evasion from cytolytic killing by NK cells and that targeting PCNA may allow the immune system to recognize and kill malignant cells more effectively.

### 7.3. T Lymphocytes

T lymphocytes are one of the key players in the adaptive immune system. There are, however, little data published about the role of PCNA within them. From our search, PCNA may have a role in the activation of T lymphocytes. In a study assessing the regulation of PCNA expression during the G1 phase of human T lymphocytes and on the relationship between PCNA expression and the rate of T-cell proliferation, PCNA expression was found to be a signal after IL-2 binding and signalled that the cell was undergoing DNA synthesis [65]. Interestingly, in both normal T lymphocytes and malignant Jurkat lymphocytes, PCNA has also been shown to be expressed and associated with cyclins and cyclin dependent kinases (CDK) 4, 5, and 6. These cyclins are key components of cell cycle machinery in driving G1 to S phase transition via phosphorylation and CDK4/6 inhibitors are currently in clinical use for treatment of metastatic breast cancer, although no significant expression patterns were seen between the two cell groups [66].

### 7.4. Macrophages

PNCA is also found within the cytoplasm of macrophages and much of published data around these cells centre around tumour-associated macrophages (TAMs). Tumour-associated macrophages undergo polarization into the M1 or M2 phenotype in response to tumour chemokines [67]. These simplistic phenotypes exert conflicting effects on tumours, either facilitating or inhibiting tumour growth. The M1 phenotype secretes pro-inflammatory cytokines such as IFN-alpha, TNF-alpha, and IL-12, eliciting an anti-tumour response. The M2 phenotype facilitates tumour growth, suppressing an immune response by excreting anti-inflammatory cytokines IL-10, IL-12, and TGF-alpha. Alongside this, M2 macrophages possess the ability to stimulate tumour growth and angiogenesis through secretion of IL-17, IL-23, fibroblast growth factors, and vascular endothelial growth factors [68]. TAMs that have a population of cytosolically expressed PCNA are associated with poor prognosis for cancer patients. The PCNA+ TAMs are specifically associated with hormone receptor negative high-grade tumours [69] and a decrease in recurrence free survival [68]. Socioeconomic status has also been linked to cancer prognosis after studying the presence of PCNA+ TAMs in Caucasian and non-Caucasian populations [70]. It was found that Hispanic women had a higher PCNA+ TAM count in breast cancers and decreased recurrence-free survival compared to Caucasian women. While this study suggests a link between an immunological response to cancer and socioeconomic status, confounding factors such as the time between disease onset and detection may have distorted the results.

When PCNA+ TAM genes were compared to those of M1 and M2 phenotypes, it was found the PCNA+ TAMs shared significantly more genes with the M1 phenotype than the M2 [68]. This was surprising given the conflicting nature between PCNA+ TAM association with poor prognosis and the anti-tumour immune response that M1 macrophages elicit. However, as the data were derived from a heterogeneous cell collection of stromal cells, tumour cells, and surrounding cells, there is no definitive conclusion that the PCNA+ TAMs are M1, but rather that the PCNA+ TAMs are associated with M1-type tumours. Alongside this, recent studies have shown that there are more than two phenotypes of TAMs and compartmentalization of TAMs into only two categories is an oversimplification of the complexities involved in phenotypic partitioning [68].

## 8. Can PCNA Overexpression Be Targeted?

Although PCNA is ubiquitously present in all cells, it is overexpressed in most cancers and associated with cancer virulence. It is found in the cytoplasm as well as around the nucleus. The association of PCNA in cancer has led to many considering the significance of its role within cancer cells. Malkas et al. identified two distinct isoforms of PCNA exist within breast cancer cells, with only one isoform existing within normal cells [5]. It has been suggested that this post translational modification of PCNA could be used as a biomarker for early detection of cancer [5]. However, reports on this are sparse and there is a suggestion that the difference in isoforms described is a combination of the different efficiencies of the anti-PCNA antibody used and the different protein levels of PCNA in cancer and normal cells [4].

Nevertheless, the appeal of a cancer-specific isoform has led to interest in a potential novel target for therapy. ATX-101 is an orally active compound that selectively targets caPCNA regulatory roles during cellular stress, and a first-in-class compound in clinical development. It has recently completed phase 1 clinical trials reporting possible dose escalation and minimal toxicity [71]. In this study, twenty-five patients with progressive, late-stage solid tumours, including breast cancer, were given weekly infusions of ATX-101 with the standard three by three dose escalation, up to a maximum dose of 60mg/m^2^. Although treatment efficacy was not the primary end point of this study, the authors note that clinical stability was achieved in 70% of patients, which is promising given the treatment resistant nature of the selected patient group. Further clinical studies have been initiated to assess efficacy and the data will provide insight into this novel target. There are now several PCNA targets under development and studies related to their efficacy and the mechanism of actions summarised in Table 1.

Given the knowledge of the role of PCNA in neutrophils and macrophages, the popularity of immunotherapy, the development of virotherapy in the current era of cancer management, and the development of a specific target for PCNA, the next natural step would be to explore a combination of treatments within cancer patients to assess if these regimes compliment.

## 9. Conclusions

The functions of PCNA are not limited to DNA replication and repair; rather, the multifunctional protein contributes to an array of cellular processes which diversify following the transition of healthy cells to unhealthy cells. This review summarises the role of PCNA in states of inflammation such as malignancy and viral infection, and discusses the currently available data about the role of immune cells within this setting. Although information is not complete, the data show that PCNA plays a key role in the function of immune cells and replication of virus and malignant cells. Suppression of PCNA may lead to an increase in innate immune function (through activation of NK cells and macrophages) in addition to a decrease in cancer cell growth (through inhibitors of DNA replication at the level of the replication fork). Cancer-specific PCNA inhibition may allow the use of oncolytic viruses alongside PCNA inhibition which could potentially unleash a new treatment modality. However, caution must be given to potential interaction as some virus, particular HSV, seem to require PCNA for their own replication. Further studies in this interesting area are needed.

## Figures and Tables

**Table 1:** A summary of PCNA inhibitors and their targets.

Name	Mechanism of Action	Key Information	References
T2AA	Blocks protein interactions between the PCNA IDCL and proteins containing the PIP-box peptide motif Inhibits PCNA–Pol δ interaction Inhibits de novo DNA synthesis	Small Molecule T2AA is a T3 analogue that exhibits almost thyroid hormone activity Sensitises cells to cisplatin chemotherapy	[43,72]
PCNA-I1	PCNA-I1 binds at the interface between PCNA monomers, stabilizes the homotrimer, and may interfere with protein–protein interactions	Small Molecule Inhibits the association of PCNA with chromatin	[73,74]
AOH1160	Small molecule target against caPCNA	Small Molecule, Oral SCID mice Tested in vivo in neuroblastoma, breast, SCLC Lacked metabolic properties to proceed to clinical studies	[6]
AOH1996	Small molecule target against caPCNA	Small Molecule Oral SCID mice Tested in vivo in neuroblastoma, breast, SCLC	[7]
p21C2	p21-PCNA interaction	Peptide No in vivo cancer testing described	[75]
p21PBP	p21-PCNA interaction	Peptide No in vivo cancer testing described	[76]
Y211F CPPP	Y211F peptide blocks phosphorylation of PCNA tyrosine 211	Peptide Inhibits TNBC cell growth of orthotopic implanted tumours	[77]
Con1-Spop	Acts directly on PCNA to cause impaired mitotic division and mitochondria dysfunction	Peptide Tested in T-REX 293 Dox-inducible system	[78]
ATX-101	Disrupts PCNA from interacting with APIM-containing proteins	Peptide Used in combination with other cancer therapeutics Immunocompetent mouse models Phase 1 trial	[71]
α-PCNA aptamer	Blocks replication of the DNA template by forming an α-PCNA aptamer/PCNA/DNA pol complex that is unable to bind the primer-template DNA	Aptamer Has been used as part of a co-delivery system with doxorubicin to target 4T1 cells in vivo	[49,79]
14-25-9	Inhibition of NKp44-PCNA immune checkpoint, as 14-25-9 is a checkpoint-blocking mAb against proliferating cell nuclear antigen (PCNA)	Monoclonal antibody Enhances cytotoxic function of NK92-NKp44-1 cells and inhibits tumour growth in PDX models	[51]
NKp44-pep8	Partly block the NKp44–PCNA interaction to target intracellular PCNA	Peptide Immunocompetent mouse models (4T1 and B16 derived) NKp44 is not expressed on mouse NK cells, so anticancer effect of NKp44-pep8 in vivo likely to not be attributed to upregulation in mouse NK cell function	[79]
mAb14	Monoclonal antibody to PCNA	Monoclonal antibody Study in cell lines of primary cutaneous T-cell lymphoma to enhance NK function	[50]

## Data Availability

No new data were created in this review.
